# COVID-19 Vaccine Hesitancy Among Various Segments of the Population in Turkey: A Literature Review

**DOI:** 10.3390/vaccines13010044

**Published:** 2025-01-07

**Authors:** Sezer Okay

**Affiliations:** Department of Vaccine Technology, Vaccine Institute, Hacettepe University, 06230 Ankara, Turkey; sezerokay@gmail.com; Tel.: +90-3123053494

**Keywords:** COVID-19, SARS-CoV-2, vaccine hesitancy, vaccine refusal

## Abstract

Vaccine hesitancy, which refers to the reluctance to be vaccinated, poses a major risk to public health in preventing infectious diseases. This hesitancy has been evident for many years, especially regarding childhood vaccines. The main factors contributing to this hesitancy include religious or personal beliefs, concerns about safety and efficacy, and desire to receive more information from healthcare providers. This literature review examines hesitancy regarding COVID-19 vaccines in different population segments in Turkey. Hesitancy rates and reasons in the general population and specific groups such as pregnant women, parents, healthcare workers and students were presented based on published research articles. Approximately half of the Turkish population declared their hesitancy towards COVID-19 vaccines. A negative correlation was found between vaccine hesitancy and health literacy. The relationship between COVID-19 vaccine hesitancy and religiosity was also investigated. Age is another factor affecting this vaccine hesitancy. Older age was shown to be correlated with positive attitude towards COVID-19 vaccination. Moreover, participants with positive attitudes towards other vaccines, those with chronic diseases and those with a personal history of COVID-19 were more likely to have positive perceptions of COVID-19 vaccines. Higher life satisfaction and non-smoking status were associated with a higher likelihood of COVID-19 vaccine acceptance. Increased scientific data on the efficacy and side effects of COVID-19 vaccines and more information from healthcare professionals would likely reduce the hesitancy towards COVID-19 vaccines.

## 1. Introduction

Vaccines have become an integral part of public health, saving huge numbers of lives. Although the majority of the world’s population is aware of the importance of vaccination and has been vaccinated, there is a growing negative attitude towards vaccines. According to the World Health Organization (WHO), vaccine hesitancy is among the top 10 factors that threats to public health worldwide. There are different degrees of reluctance to be vaccinated. Vaccine hesitancy refers to experiencing a period of uncertainty and indecision about vaccination before deciding whether to be vaccinated. People who are vaccine-hesitant have reservations about certain aspects of vaccines, and their opinions may change. However, vaccine refusal is a stronger negative attitude towards vaccines compared to hesitancy. People with this attitude are determined to completely refuse to be vaccinated and cling to that idea [1,2].

The COVID-19 pandemic created an urgent need for vaccines. Therefore, the paradigm of vaccine development had to be modified, and clinical phase studies were completed in a shorter time than the classical vaccine development process [3]. This acceleration in the commercialization of the COVID-19 vaccines raised questions about their safety and efficacy. A survey of 23,000 adults from 23 countries conducted between June and July 2022 reported that concerns about the safety and efficacy of COVID-19 vaccines were strongly associated with vaccine hesitancy [4].

**Figure 1:**
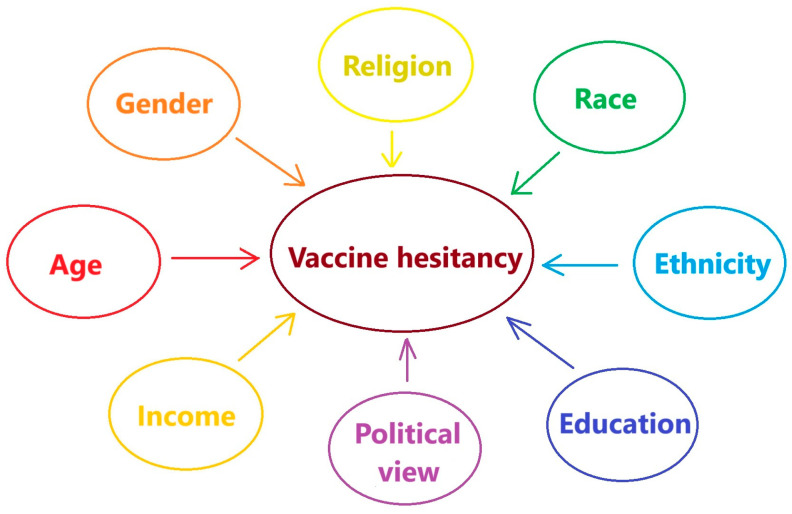
Leading factors having an effect on the vaccine hesitancy [5].

Factors affecting vaccine hesitancy include gender, age, religion, race/ethnicity, education, political view, and income (Figure 1) [5]. The impact of these factors may change in different groups. Additionally, conspiracy theories are important determinants that trigger vaccine hesitancy. Physicians play a crucial role in shaping vaccination decisions, and their response to conspiracy theories can significantly influence public attitudes. A survey study among physicians in Germany assessed the relationship between belief in conspiracy theories that SARS-CoV-2 is a hoax or man-made and attitudes toward COVID-19 vaccines. It was observed that physicians who believed in these theories were less likely to accept and recommend COVID-19 vaccines [6]. Another study in Nigeria found that 26.4% of healthcare workers (HCWs) included in the study believed that COVID-19 vaccines contained digital microchips, while 30% believed that the vaccines could alter a person’s DNA [7]. Believing in unrealistic conspiracy theories can lead to vaccine hesitancy among HCWs and negatively affect the views of patients, so this factor should be taken into consideration in situations that threaten public health, such as pandemics.

The association between conspiracy theories and COVID-19 vaccine acceptance was also investigated in the general population of China. Conspiracy beliefs were found to directly and indirectly affect vaccine hesitancy. The indirect effect was determined through medical mistrust, knowledge, confidence and indifference to vaccines [8]. Social media applications on the internet play an important role in the spread of conspiracy theories. A study conducted in Japan investigated the relationship between the use of different types of media (newspapers, television or radio, internet or news applications, social networking services) and COVID-19 vaccine hesitancy. A significant relationship was shown between vaccine hesitancy and the use of the internet or news applications, especially in middle-aged and elderly groups [9].

Religion has also been identified as an important factor influencing vaccine hesitancy. In a study investigating the interaction between religious beliefs and hesitancy towards COVID-19 vaccines in 15 countries in Africa and Asia Pacific, eight religious groups (African Christian, African Muslim, Asian Christian, Asian Muslim, Animist, Buddhist, Hindu, and Atheist) were surveyed at two different times (Wave 1 and Wave 2). The lowest hesitancy was determined among Atheists and Buddhists in Wave 1 and among Atheists and Muslims in Wave 2. The link between religion and vaccine hesitancy was reported to be complex and context-dependent. The effects of time and educational status on vaccine acceptance were found to be heterogeneous [10].

Different populations have their own dynamics regarding vaccine acceptance and hesitancy. Therefore, studies on attitudes towards COVID-19 vaccines in the Turkish population were examined here. Vaccine hesitancy in the general population as well as specific groups such as HCWs, pregnant women, parents for their children, and university students were considered separately.

## 2. Vaccine Hesitancy in the General Population

Many studies have shown that about half of the Turkish population had negative attitudes towards COVID-19 vaccines, and that the situation was not changed before and after the implementation of widespread vaccination, but there are also studies to the contrary. Increased health literacy and health perception were found to be associated with more positive attitudes towards vaccines (Table 1). Participants who were mostly male, older, and had more years of education were more likely to be vaccinated. The link between religiosity and vaccine hesitancy was controversial.

### 2.1. The Rate of Vaccine Hesitancy

In a meta-analysis aimed to assess COVID-19 vaccine hesitancy in Turkey, analyzing a total population of 15,164 individuals, the overall vaccine hesitancy rate was found to be 30.5% (24.3–36.8%), with a prevalence of 39.8% (31.4–48.2%) prior to initiation of vaccination on 14 January 2021 [23]. A study conducted between October 2020 and January 2021 involving 2023 participants reported that 31.3% of participants were not willing to receive the COVID-19 vaccine [17]. In two studies performed in December 2020, 42.9% of 1098 participants [15] and 45.3% of 384 participants [24] expressed their hesitation about the COVID-19 vaccines. Additionally, a study conducted between July and December 2020 reported that vaccine refusal was 21.5%, and hesitancy was 29% among 3888 adult participants. However, an increase in vaccine hesitancy/refusal was found from 43.9% to 58.9% in parallel with the increase in the spread of COVID-19 [11].

A meta-analysis by Gulle et al. showed that the vaccine hesitancy rate decreased to 20.4% (12.9–28%) after the introduction of widespread vaccination. It was suggested that the decrease in vaccine hesitancy may be due to regulatory approvals, publications showing the benefits of the vaccines, positive news from other countries, and promising results in vaccinated individuals [23]. However, many studies from Turkey reported high rates of hesitation towards the COVID-19 vaccines in this period. A comprehensive study including 4539 participants representing the different regions of Turkey reported that the rate of hesitation was 58.4% about COVID-19 vaccination [25]. Likewise, Baysal and Demirkol found that 54.9% of 502 participants hesitated about vaccination, and 7.2% outright refused the vaccine [20]. A cross-sectional study conducted in June 2021 in Istanbul with 8675 participants showed that 45.6% of the participants expressed hesitancy about COVID-19 vaccines [16]. Additionally, a survey conducted in April 2021, involving 1009 adult participants, revealed that 37.3% of participants held negative perceptions of COVID-19 vaccines [18].

### 2.2. Factors Associated with Vaccine Hesitancy

#### 2.2.1. Health-Related Issues

Various studies showed a significant association between health literacy and attitudes towards vaccines. Kocaay et al. [12] reported that the health literacy score was significantly lower (*p* = 0.008) in the group of participants who refused to receive COVID-19 vaccines (7.5% of 496 participants) or were hesitant about being vaccinated (14.3% of 496 participants). Similarly, Sayar et al. [13] found the health literacy score significantly lower (*p* = 0.047) in the group of unvaccinated participants (17.6% of 334 participants). Also, Kilic et al. [14] found a positive correlation (*p* = 0.019) between positive attitude towards COVID-19 vaccines (approximately 30% of the participants) and increased level of health perception. In addition, Kuçukkarapinar et al. reported that reduced knowledge about disease prevention as well as decreased perception of disease risk and overloaded health system were associated with vaccine refusal and hesitancy [11].

Concern about side effects was an important reason for hesitancy about COVID-19 vaccines. In a study conducted by Tosun et al., 42.6% of the participants stated that they would not be willing to have the COVID-19 vaccine if it had side effects [15]. Moreover, Atac et al. reported that 97.5% of the hesitant participants and 73.0% of the participants willing to receive the COVID-19 vaccine were concerned about side effects [16].

Higher life satisfaction [14] and non-smoking status were linked with an increased probability of COVID-19 vaccine acceptance [17]. Moreover, participants with a positive attitude to other vaccines [15,17,18,19], those with chronic diseases [11,13,15,17,20] and the ones with a personal history of COVID-19 [14,18] were more likely to harbor positive perceptions regarding COVID-19 vaccines.

Effect of mental health on the attitudes towards vaccination was also investigated. Increased concern about declining mental health was shown to be associated with higher vaccine acceptance (*p* < 0.001) [11]. Also, higher psychological reactance (resistance to external influence) was found to be related to lower vaccination intention (54.7% of the participants), while higher collectivism (prioritizing group benefits over individual success) was associated with a greater intention to be vaccinated [21].

#### 2.2.2. Age

Age is another factor affecting COVID-19 vaccine hesitancy. Köse et al. found the highest vaccine acceptance and lowest rejection in the age group of 50–59 years, followed by the age group of 60+ years [17]. Furthermore, the lowest vaccination rate (80.0%) was reported in individuals aged 39 years and younger, while the highest rate (92.3%) was in the age group of 60–65 years [13]. Additionally, Baysal et al. showed that vaccine hesitancy was less in participants aged 65 years and above [20]. Similarly, the highest rate of willingness to receive COVID-19 vaccines (58.1%) was in the age group of 51+ years in the study performed by Tosun et al. [15]. Kilic et al. also reported that increasing age was significantly associated with willingness to be vaccinated against COVID-19 (*p* = 0.037) [14]. On the other hand, in a study conducted by Sonmezer et al., individuals aged 30 years and above were less likely to have positive perceptions towards COVID-19 vaccines; however, the rate of participants aged 50–59 and 60+ was 6% and 0.9%, respectively [18].

#### 2.2.3. Gender

One of the predictors of vaccine refusal included gender in many studies. Kuçukkarapinar et al. showed that the vaccine refusal and hesitancy rates, 23.3% and 31.5%, respectively, were significantly higher in females (*p* < 0.001) [11]. Kilic et al. also reported that positive attitude score for COVID-19 vaccine was significantly lower in females (*p* < 0.001) [14]. Females exhibited higher rates of vaccine rejection and refusal in other studies at a rate of 69.5% [15,20]. However, Sonmezer et al. [18] and Karakaş et al. [22] reported that the negative attitude towards COVID-19 vaccines was significantly higher in males (*p* = 0.026 and *p* < 0.001, respectively). On the other hand, Köse et al. [17] and Sayar et al. [13] did not find a significant difference between males and females in terms of attitudes towards vaccination.

#### 2.2.4. Religiosity

The relationship between COVID-19 vaccine hesitancy and religiosity has also been examined. In a study conducted in Turkey, where the majority of the population is Muslim, participants (*n* = 570, median age = 26.69, 69.8% female) who identified themselves as religious reported less trust in science and generally had more negative attitudes towards vaccination than non-religious participants [19]. However, in a cross-sectional study of 1046 adults, 35.1% of participants had not been vaccinated against COVID-19, and religiosity scores were lower in the unvaccinated group [22]. Moreover, another study found no significant association between COVID-19 acceptance/avoidance and religious attitudes [14]. This inconsistency may be due to the nature of the samples and scales used in the studies, as well as the wide range of perceptions of religiosity.

#### 2.2.5. Education

An increase in the number of education years was identified as a negative predictor for vaccine hesitancy. Kuçukkarapinar et al. compared the attitudes towards COVID-19 vaccines between participants with ≤12 years and ≥13 years of education and showed that the former group was more negative towards the vaccines [11]. According to Sonmezer et al., participants with ≤secondary school education had more negative perceptions of COVID-19 vaccination than those with ≥bachelor’s degrees (*p* = 0.003) [18]. It was also reported that education year and vaccine refusal scores were negatively correlated [12] and higher mistrust of vaccine benefit was found among primary school graduates [16]. However, higher rates of vaccine rejection and refusal were shown among university graduates by Baysal and Demirkol [20]. On the other hand, Tosun et al. did not find a significant difference between the education levels and negative attitudes towards COVID-19 vaccines [15].

### 2.3. Multinational Studies Including Turkey

Multinational studies on hesitancy towards COVID-19 vaccines were also conducted. In a study performed via online survey in the United Kingdom (UK) (*n* = 1088) and Turkey (*n* = 3936), vaccine hesitancy was found to be 31% in Turkish participants and 14% in UK participants. Vaccine refusal was found to be 3% in both countries. Furthermore, 54% and 63% of Turkish and UK participants, respectively, believed that SARS-CoV-2 was of natural origin, significantly increasing the likelihood of accepting the COVID-19 vaccine [26]. In another study with participants from the United Kingdom (*n* = 1533), the United States (US) (*n* = 1550), and Turkey (*n* = 1567), vaccine acceptance rates were found to be 82.9%, 61.2%, and 47.2%, respectively. Vaccination of a trusted contact and a recent death from COVID-19 were reported to encourage vaccination in all three countries. However, individuals who initially refused to be vaccinated (6.1%, 20.5%, and 11.6%, respectively) were less affected by these factors than those who were hesitant (11.0%, 18.3%, and 41.2%, respectively). Turkey was the country with the highest vaccine hesitancy and also had the highest average effectiveness scores of incentives. Therefore, it was suggested that a conscious strategy to encourage vaccination could reduce hesitancy in Turkey [27].

## 3. Vaccine Hesitancy Among Pregnant Women

In a study conducted by Miral et al. in Istanbul, Turkey, including pregnant and lactating women, 65.6% of 360 participants had a positive attitude towards the COVID-19 vaccine [28]. However, another study in Turkey including 211 pregnant women reported a vaccination rate of 58.3% (*n* = 124). The results showed that factors affecting vaccine hesitancy in pregnant women were the spouse’s education level, gestational age and number of pregnancies, presence of chronic diseases in the family, and pre-history of abortions [29]. In addition, another study conducted with 561 pregnant women reported that vaccine hesitancy increased in pregnant women with high income (*p* < 0.001) and lack of appropriate advice (*p* = 0.015). The primary reason for vaccine hesitancy was fear of side effects, which was seen in 62.7% of unvaccinated pregnant women. Interestingly, 83.8% of participants had received a tetanus vaccine [30].

A qualitative content analysis was conducted on 16 women about their hesitancy to receive COVID-19 vaccination during pregnancy. The vaccine hesitancy rate was 75%, and the main reason was fear of possible side effects of vaccines on babies and complications such as premature birth or miscarriage. However, trust in vaccines, provided by information from reliable sources and social support, created a positive perception of pregnant women about the decision to receive the vaccine. In addition, fear of severe COVID-19 caused a positive attitude towards vaccination [31].

Vaccine hesitancy rates in pregnant women were similar to the general population. Despite a high rate of positive response to the tetanus vaccine, higher hesitancy towards COVID-19 vaccines was primarily due to fear of possible side effects, particularly those that could affect the baby. Increasing data on the safety of COVID-19 vaccines for pregnant women may reduce hesitancy.

## 4. Vaccine Hesitancy of Parents for Their Children

Studies examining parents’ perceptions of their children’s COVID-19 vaccination have shown that parents’ attitudes toward vaccination determine their hesitancy to vaccinate their children [32]. In a cross-sectional study, 59.9% of 1035 participants were eager to get COVID-19 vaccine for themselves, while only 36.3% were positive about their children being vaccinated. Interestingly, 83.9% of parents were willing to have their children vaccinated if a SARS-CoV-2 variant with high mortality rates emerged in children. HCWs were more likely to accept vaccination of their children compared to non-HCWs [33]. In another study, the vaccination rate of the 819 parents was 70.3%, and 69.0% of the parents were positive for the vaccination of their children [34]. The rate of parents’ willingness towards vaccination of their children was found to be high in that single-center study probably due to the nature of sample as the participants were from a city where vaccination rates were high (86.8%) according to the Ministry of Health of the Republic of Turkey [35]. The authors also found high rates of awareness about COVID-19 vaccines (88.3%), and the necessity of vaccination for the COVID-19 pandemic (89.4%).

In a cross-sectional study conducted with 1087 parents, it was reported that age, income, education and marital status had no significant association with the willingness to receive the COVID-19 vaccine. However, it was found that men had more positive attitudes towards vaccination compared to women (*p* = 0.022). In addition, having a family member with COVID-19 history was significantly associated with an increase in positive vaccine attitudes (*p* = 0.007). Receiving a COVID-19 vaccine, experiencing vaccine-related side effects, and losing a family member to COVID-19 were not significantly associated with positive attitudes. However, not being hesitant regarding childhood vaccines was found to be associated with an increase in positive attitudes towards COVID-19 vaccines (*p* = 0.001). The majority (70.14%) of parents stated that they had insufficient information about childhood vaccines. Additionally, the social media had a negative influence on the parents towards COVID-19 vaccines (*p* = 0.007) [36]. Another study reported that websites (34.27%) and social media (24.51%) were common reasons for refusing the COVID-19 vaccines among 103 parents who had refused the Hepatitis B vaccine for their children in the last decade [37].

In a study of parents and adolescents examined in a child psychiatry outpatient clinic (*n* = 248), 7.3% of parents and 7.7% of adolescents were reported to be hesitant, with 1.6% and 2.8% against vaccination, respectively. In addition, adolescents with anxiety disorders had a higher vaccination rate (60.8%), and the number of unvaccinated participants was higher among those without a psychiatric diagnosis (69.4%). Patient age, vaccine hesitancy, and parental vaccination status and chronic disease status in a family member were reported as predictive factors for adolescent vaccination [38].

In studies that included participants with higher health perceptions, it was observed that parents were less hesitant about vaccinating their children against COVID-19. In addition, parents showed a protective attitude that increased their willingness to get their children vaccinated against a SARS-CoV-2 variant with higher virulence. Attitudes towards childhood vaccines and COVID-19 vaccines were in the same direction. Moreover, social media contributed negatively to parental vaccine hesitancy. Given the high childhood vaccination rates in Turkey, hesitancy towards COVID-19 vaccines may be explained by parents’ safety concerns. Therefore, authorities need to report the current status of COVID-19 vaccines for children, especially through pediatricians.

## 5. Vaccine Hesitancy Among Healthcare Workers

A study conducted with 1808 HCWs before COVID-19 vaccination began found that anxiety about the adverse effects of COVID-19 vaccines was higher in women and those aged 36–50, and that doctors, nurses, midwives, those who were married, those with children, and those who followed WHO’s scientific publications and announcements were more likely to accept the COVID-19 vaccine. Although most HCWs stated that vaccinating people around them, doctors, ministers, or senior officials would reduce COVID-19 vaccine hesitancy, 19% of participants stated that their vaccine hesitancy would continue. Moreover, 4.1% of the HCWs had a strong negative attitude towards all vaccines. Interestingly, more than half of the HCWs disagreed that the COVID-19 vaccine should be mandatory. Among the participants, 15.6% had parental vaccine refusal and 31.9% had hesitancy. Possible short-term and long-term side effects of vaccines caused concern on 56.2% and 79.7% of HCWs, respectively. Distrust of COVID-19 vaccines was attributed not only to pharmaceutical companies, but also to countries previously known to produce low-quality products. Health authorities and academics were also linked to vaccine hesitancy due to unconvincing, contradictory or unclear statements [39].

In a study conducted with 126 physicians and 150 midwives/nurses before the start of vaccination, it was reported that 50.4% of HCWs had a positive view on receiving the COVID-19 vaccine, 29% were hesitant, and 20.7% had a negative view. It was determined that the vaccine acceptance rate was significantly higher (*p* < 0.001) among physicians (65.9%) and men (79.2%). This study also revealed that HCWs were concerned about the possible short-term and long-term side effects of the vaccines at a rate of 45.6% and 63.7%, respectively [40]. In another study conducted before the start of vaccination, 72.6% of 442 HCWs stated their positive attitude towards the COVID-19 vaccines. Interestingly, only 55.9% of HCWs believed that the COVID-19 vaccine would be a factor that would end the pandemic. Differences in attitudes towards the vaccine were observed between HCWs aged over 41 and under 30. A significant and positive relationship was shown between the participants’ attitudes towards the measures taken regarding COVID-19 infection and their attitudes towards the vaccine. Unknown long-term side effects of the vaccine caused vaccine hesitancy [41].

In a study conducted with 806 midwives before vaccination started, only 16.8% of the participants stated that they considered getting the COVID-19 vaccine, 48.8% considered getting vaccinated after the doubts about vaccine safety were eliminated and 10.5% refused to be vaccinated. The main reasons for reluctance to be vaccinated were the lack of sufficient clinical studies (75.6%) and the belief that imported COVID-19 vaccines were not adequately controlled (48.1%) [42].

The rate of vaccination was found to be much higher in the studies conducted after vaccination campaign than the ones before it. In a study including 378 HCWs, vaccination rate was 93.7% for at least one dose and 86.8% stated that they were fully vaccinated. A significant association was observed between not being fully vaccinated and being a non-physician HCW (*p* = 0.001) and having a history of SARS-CoV-2 infection (*p* < 0.001) [43]. In another study conducted with dentists (*n* = 458) in April 2021, 12.9% of the participants were not vaccinated against COVID-19, while 87.1% of the participants declared that they were vaccinated. The rate of vaccination was significantly higher in men (93.2%, *p* = 0.019) and married people (89.9%, *p* = 0.011) [44].

In studies conducted with HCWs, it is noteworthy that there are significant differences in vaccine hesitancy and vaccination rates between studies conducted before and after widespread vaccination began in January 2021. While vaccine hesitancy rates were reported to be high before vaccination began, subsequent studies found that vaccination rates increased. The vaccination rate among HCWs was higher than the general population, and among HCWs, physicians were more likely to receive the COVID-19 vaccine. The main reason for the negative attitudes among HCWs was uncertainties about COVID-19 vaccines (safety, short-term and long-term side effects, etc.).

## 6. Vaccine Hesitancy Among University Students

A study conducted with 602 university students found that the vaccination rate was 89.7%. It was also observed that 72.96% of vaccinated students had two doses of the vaccine, and 59.5% were skeptical about COVID-19 vaccines. Male students had significantly higher positive attitudes towards the vaccines (71.7%, *p* = 0.002). There was a significant relationship between receiving expert information and students’ attitudes towards vaccines (*p* < 0.001), but it did not matter which type of healthcare professional the information was received from (*p* = 0.159). The majority of students who had no idea about vaccines did not receive expert information. Surprisingly, participants who received information (53.6%) had a mostly negative view of vaccines, while those who did not receive information (52.8%) had a mostly positive view [45].

The rate of vaccination was found to be 96.6% in another study conducted with 388 university students. However, 50% of the participants expressed their hesitancy about COVID-19 vaccines due to the varying views of healthcare professionals. In addition, the majority of students (64.7%) stated that their confidence would not increase even if the vaccine was produced domestically, and 64.9% stated that they did not think the vaccine caused infertility [46]. Interestingly, a cross-sectional study conducted on 498 nursing students reported that 64.5% of participants stated their eagerness to have the COVID-19 vaccine, which is a low rate for future HCWs. Their concerns included the insufficient information about COVID-19 vaccines (65.7%), vaccine safety (45.8%) and effectiveness (41.6%). Income, education, previous vaccine refusal, vaccine efficacy and side effects, and the false belief that the vaccine has an effect on genetics were predictors of vaccine hesitation [47].

University students had a higher COVID-19 vaccination rate compared to the general population, despite having the same hesitancy rate. It is noteworthy that receiving expert knowledge increased negative attitudes towards the vaccines. The high hesitancy rate among nursing students also suggested that high perceptions of safety concerns and uncertainties about COVID-19 vaccines increased hesitancy among university students.

## 7. Vaccine Hesitancy Among Immigrants

In a study conducted in Munich among Turkish- and German-speaking patients with immigrant backgrounds, 58.3% of 420 participants preferred the Turkish questionnaire, 82.9% had an immigrant background, and 47.9% were considering vaccination. Of the participants with an immigrant background, 42.3% were considering vaccination, compared to 76.5% for non-immigrant Germans. Factors found to be positively associated with vaccinations were non-immigrant status, male gender, years of education, and age. The two main reasons for the negative attitude were insecurity/security concerns (15.3%) and lack of sufficient work (9.5%) [48].

A study conducted at the Extended Migrant Health Center in Istanbul with 571 participants found that a total of 55% of Syrian immigrants had not received any COVID-19 vaccination. The immigrants had very poor health literacy (68.7% insufficient and 20.7% limited) while it was higher in the vaccinated participants compared to unvaccinated ones (*p* = 0.025). Limited health literacy may have led to a reduced perception of the necessity of vaccination. Additionally, positive attitudes towards COVID-19 vaccines were significantly higher in men (*p* = 0.012), the elderly (*p* = 0.002), those with middle/high income (*p* = 0.035), and those with a family member with a chronic disease (*p* = 0.001) [49].

It is interesting that the rate and factors of vaccine hesitancy in Turkish immigrants was similar to that in Turkey. Although the rate of vaccination and the hesitancy-related factors were similar in the immigrants in Turkey, it is remarkable that the health literacy was very limited in this group.

## 8. Limitations and Directions for Future Research

This literature review has some limitations. First, the number of studies conducted on the general population was the highest; however, those conducted on subgroups (pregnant women, immigrants, etc.) were limited. More future studies are needed, especially on immigrants. Second, the results of the studies vary depending on the time and place of data collection because the COVID-19 pandemic was a dynamic period and the hesitancy rate was not homogeneous across Turkey. These issues should be taken into account in future studies. In addition, knowledge about the safety and efficacy of vaccines significantly affects vaccine hesitancy. Therefore, the attitude rates towards vaccines can be investigated in the same sample of participants before and after they were given sufficient information about their concerns.

## Figures and Tables

**Table 1:** The main factors associated with attitudes towards COVID-19 vaccines in general population in Turkey.

Factors	References
Health-related issues	
Health literacy	[11,12,13,14]
Concerns about side-effects	[15,16]
Life satisfaction	[14]
Non-smoking	[17]
Attitude to other vaccines	[15,17,18,19]
Having chronic diseases	[11,13,15,17,20]
Personal history of COVID-19	[14,18]
Mental health	[11,21]
Age	[13,14,15,17,18,20]
Gender	[11,14,15,20,22]
Religiosity	[19,22]
Education	[11,12,16,18,20]
